# Thyroid Function in the Time of COVID-19: A Systematic Review of Disease Progression and Vaccination Effect

**DOI:** 10.5812/ijem-146857

**Published:** 2024-10-27

**Authors:** Maryam Zarkesh, Maryam Sanoie, Shabnam Heydarzadeh, Raziyeh Abooshahab, Afsoon Daneshafrooz, Farhad Hosseinpanah, Mehdi Hedayati

**Affiliations:** 1 Cellular and Molecular Endocrine Research Center, Research Institute for Endocrine Sciences, Shahid Beheshti University of Medical Sciences, Tehran, Iran; 2 Curtin Medical School, Curtin University, Bentley, Australia; 3 Obesity Research Center, Research Institute for Endocrine Sciences, Shahid Beheshti University, Tehran, Iran

**Keywords:** Thyroid Hormones, Thyroid Diseases, SARS-CoV-2 Virus, Vaccines

## Abstract

**Objectives:**

This systematic review sought to address three key questions: (1) what differences in abnormal thyroid function test results are observed between COVID-19 patients and healthy individuals? (2) How does the severity of COVID-19 infection influence the development of thyroid dysfunction? (3) What impact do COVID-19 vaccines have on thyroid function and autoimmune processes?

**Methods:**

A literature search was conducted in PubMed, Web of Science, and Scopus from December 2019 to April 2023 to identify studies on thyroid dysfunction in COVID-19 patients without pre-existing thyroid conditions. The search focused on observational and case-control studies.

**Results:**

The literature search yielded 329 reports, from which duplicates and unrelated publications were excluded. Ultimately, 21 studies met the inclusion criteria and were selected for review. A second literature search yielded 605 reports, from which 5 studies were selected for inclusion in the systematic review.

**Conclusions:**

The findings suggest that SARS-CoV-2 infection can induce transient and reversible thyroid dysfunction, possibly through direct viral effects on the thyroid gland or via indirect immune-mediated mechanisms. Clinicians should be mindful of the potential, albeit rare, thyroid-related adverse effects of COVID-19 vaccines and monitor thyroid function, particularly in high-risk individuals.

## 1. Context

**Figure 1.**
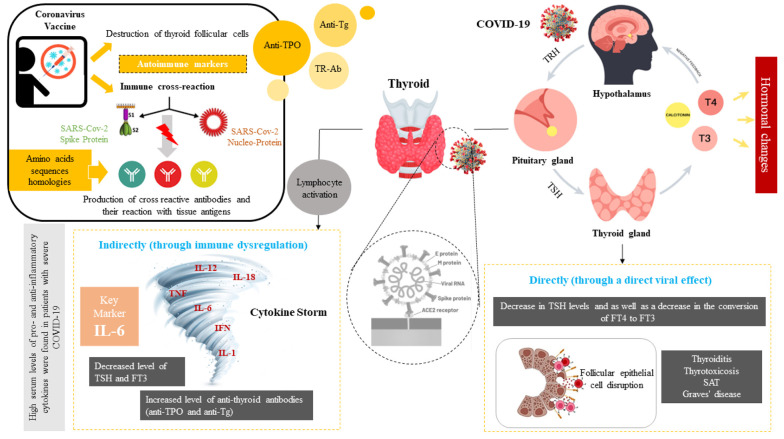
The impact of COVID-19 and vaccine on thyroid function, SARS-CoV-2 can cause hormonal changes by directly affecting the pituitary axis. On the other hand, this virus can interfere with the function of these cells by binding to the angiotensin-converting enzyme 2 (ACE2) in thyroid cells. Changes in the levels of thyroid hormones based on the direct and indirect effect of viral infection can cause various systemic changes such as the activation of various pro-inflammatory cytokines, as a result, the cytokine storm with the combined effect on the hypothalamic-pituitary axis (HPT) leads to a decrease in the level of hormones. Covid-19 vaccines destroy thyroid follicular cells by binding spike protein to angiotensin-converting enzyme II (ACE-2) receptors, leading to thyroid dysfunction and hormonal changes. Also, some homologies between the amino acid sequences of the virus with human tissue proteins such as thyroid peroxidase (TPO) leads to cross-react.

The SARS-CoV-2 virus, which causes COVID-19, can lead to multiorgan dysfunction through pulmonary and systemic inflammation. Upon infection, the virus can induce multiple organ failures via both direct and indirect pathways (Figure 1). Recent findings suggest that COVID-19 affects various organs, including the endocrine glands, via several mechanisms. However, studies on the impact of COVID-19 on the endocrine system have yielded conflicting results, complicating our understanding of its effects (1, 2).

Research indicates that the virus can affect thyroid gland function. COVID-19-related thyroid disorders have been observed in three forms: (1) hypothyroidism, (2) hyperthyroidism (thyrotoxicosis), and (3) non-thyroidal illness syndrome (NTIS), also known as sick euthyroid syndrome (SES) (3). The thyroid gland produces three main hormones: (1) thyroxine (T4), (2) triiodothyronine (T3), and (3) calcitonin. These hormones play a vital role in regulating metabolism, growth, and development in the body. The secretion of thyroid hormones is controlled by thyroid-stimulating hormone (TSH), which is produced by the pituitary gland and regulated by thyrotropin-releasing hormone (TRH) from the hypothalamus. COVID-19 infection can disrupt the hypothalamic-pituitary-thyroid (HPT) axis, leading to impaired thyroid function (4).

Additionally, studies have demonstrated that severe COVID-19 cases may trigger a cytokine storm, characterized by an overactive T lymphocyte immune response and elevated levels of pro-inflammatory cytokines, such as interleukin 6 (IL-6) and tumor necrosis factor-alpha (TNF-α) (5). This cytokine response appears to mirror the immune activation associated with inflammatory thyroid diseases. Understanding the role of immune system regulation in COVID-19 progression emphasizes the need for further evidence to clarify the relationship between thyroid hormones (THs) and COVID-19 outcomes (6).

In some studies, thyroid dysfunction has been observed in COVID-19 patients. Conditions such as Graves' disease and subacute thyroiditis (SAT) may lead to hyperthyroidism during or after SARS-CoV-2 infection (7). Moreover, the virus's impact on the HPT axis may result in central hypothyroidism, with pituitary damage emerging as a critical factor in secondary hypothyroidism, either functional or organic (8).

In light of this, numerous studies have sought to clarify the relationship between thyroid function abnormalities and COVID-19 outcomes, but the association remains unclear due to conflicting findings. Additionally, concerns have been raised regarding the potential immunological side effects of SARS-CoV-2 vaccines on the thyroid, as they may trigger inflammatory autoimmune responses that affect thyroid function. 

## 2. Objectives

This review aims to address three key research questions: First, what are the abnormal thyroid function test results in COVID-19 patients compared to healthy controls? Second, how does the severity of COVID-19 infection influence thyroid dysfunction? Third, what is the impact of COVID-19 vaccines on thyroid function and autoimmunity?

## 3. Methods

The protocol for this systematic review was conducted based on the preferred reporting items for systematic reviews (PRISMA) and registered in PROSPERO International Prospective Register of Systematic Reviews (CRD42023432969). 

### 3.1. Search Strategies and Study Selection

In this study, two separate searches were conducted as follows to answer the research questions. 

### 3.2. Systematic Review on Thyroid Function in COVID-19 Patients and Its Relationship with the Severity of COVID-19 Infection 

**Figure 2.**
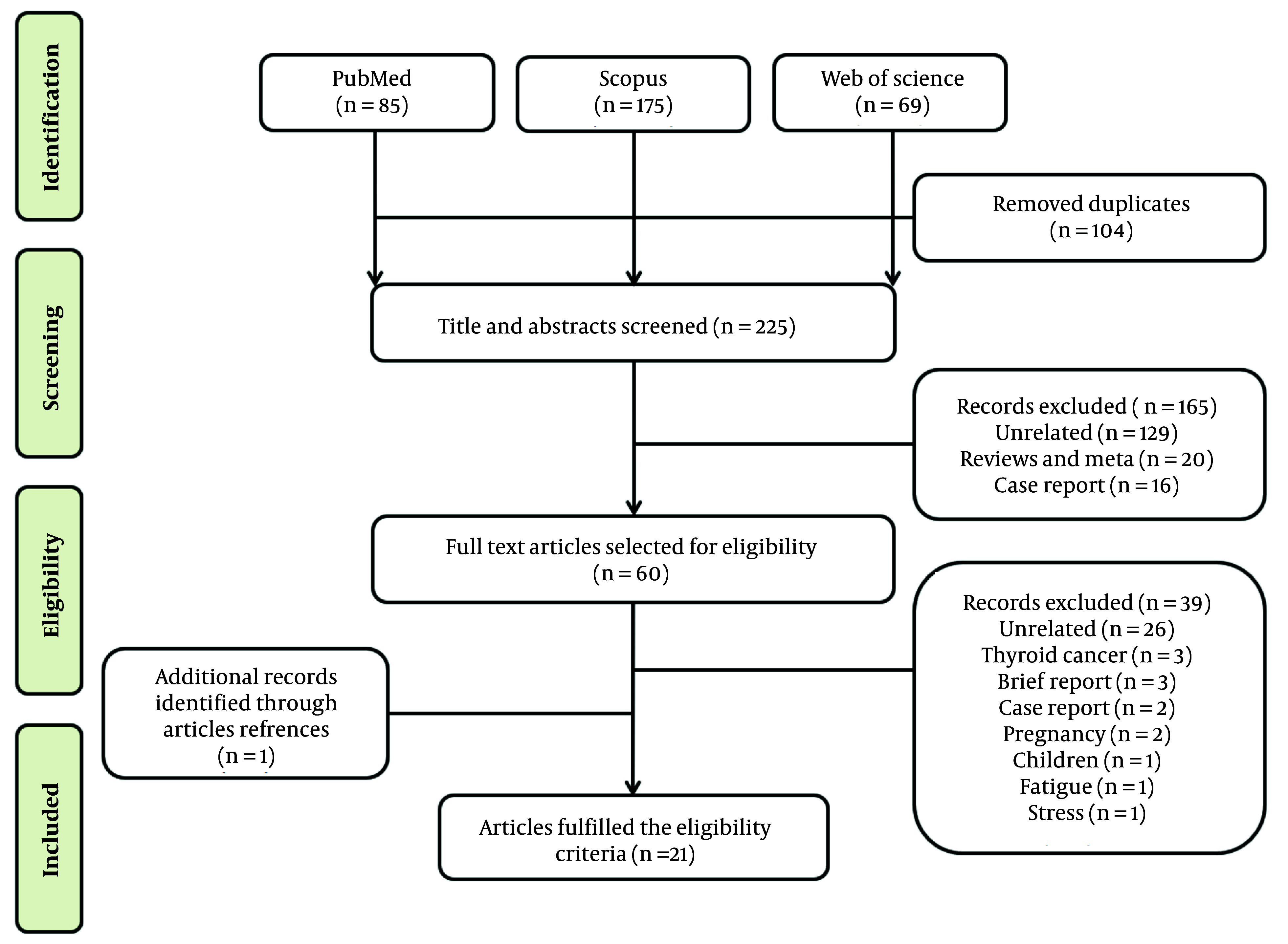
Flow chart showing the selection process for the systematic review of eligible studies on thyroid dysfunction in COVID-19 patients.

A literature search was conducted in databases including PubMed, Scopus, and Web of Science from December 2019 to April 2023, following PRISMA guidelines (9), to identify studies investigating the abnormal thyroid function test results in COVID-19 patients compared to healthy controls. Search terms included ‘COVID-19’, ‘SARS-CoV-2’, ‘coronavirus’, 'thyroiditis’, ‘thyrotoxicosis’, ‘thyroid’, and ‘thyroid disorder’. Articles eligible for inclusion were observational cohort studies and case-control studies. The initial search yielded 329 reports. After removing duplicates and unrelated publications, 165 articles were excluded and did not meet the inclusion criteria. The remaining 60 articles were fully read and independently evaluated, and finally, 21 studies were included in this systematic review (Figure 2). 

### 3.3. Systematic Review on the Effect of COVID-19 Vaccines on Thyroid Function 

**Figure 3.**
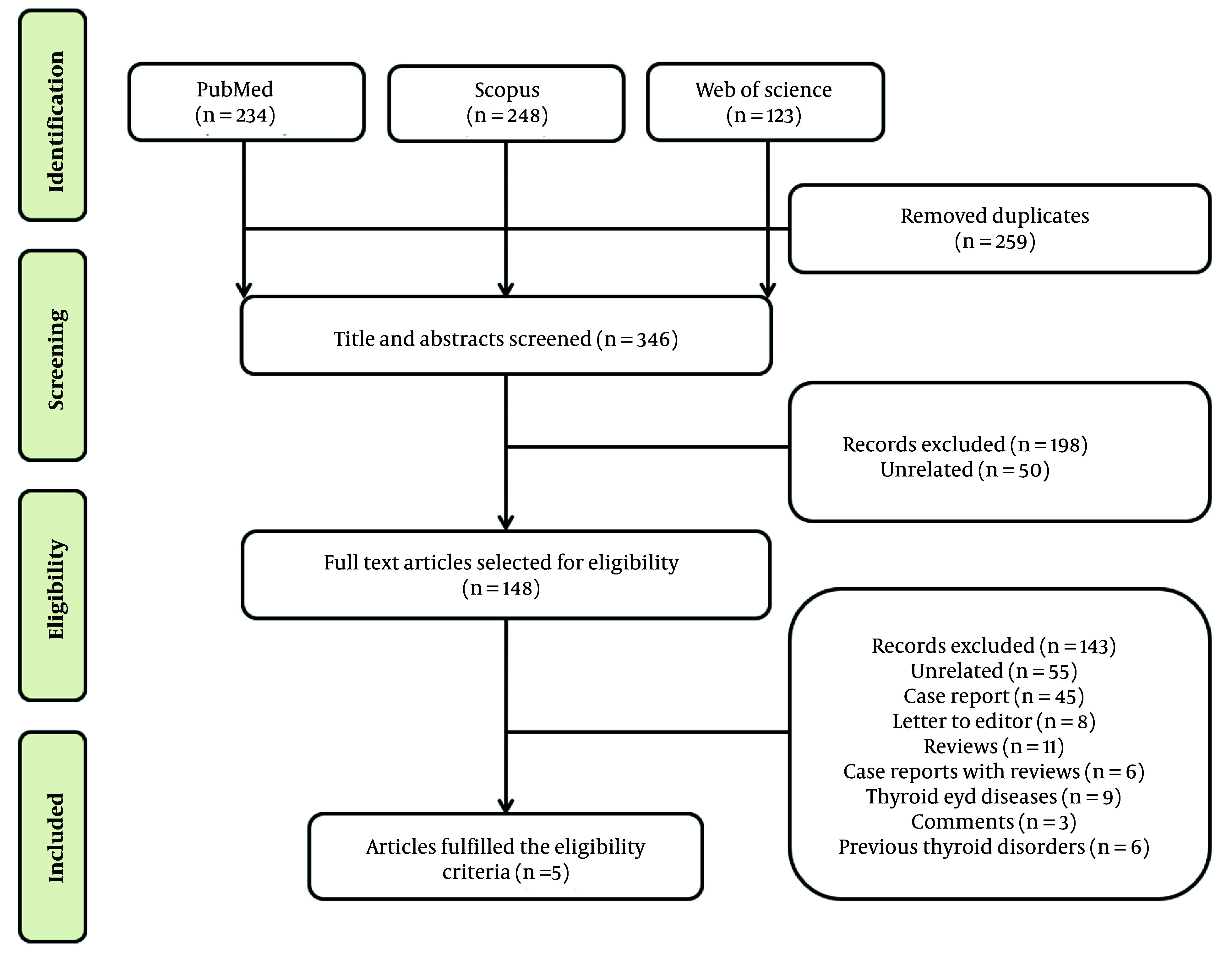
Flow chart showing the selection process for the systematic review of eligible studies on thyroid dysfunction with COVID-19 vaccine.

A separate literature search was conducted in the PubMed, Scopus, and Web of Science databases to investigate the effect of COVID-19 vaccines on thyroid function and autoimmunity. The search terms included ‘thyroid’ AND ‘Covid-19 OR SARS-CoV-2’ AND ‘vaccine’. The initial search yielded 605 reports. After removing duplicates and irrelevant publications, 198 articles were excluded as they did not meet the inclusion criteria. The remaining 148 articles were thoroughly read and independently evaluated, and finally, 5 studies were included in this systematic review (Figure 3). 

This literature review was limited to articles published in English. Data were extracted from the articles’ texts, tables, and figures. The ‘related articles’ feature and hand-searched reference lists of the included articles. were used to expand the search and obtain additional studies. The EndNote X7 tool was used for importing and managing abstracts and full texts. After the initial evaluation of the papers, duplicates were removed. Two reviewers independently (M. Z. and M. S.) undertook a two‐step selection process, with studies screened via titles and abstracts, followed by a full‐text review. All authors approved this selection process. 

Inclusion and exclusion criteria: In the first systematic review, case-control studies investigating the impact of COVID-19 infection on abnormal thyroid function tests (TFTs) in patients without pre-existing thyroid diseases were included, while observational cohort studies and case–control studies involving patients with previous thyroid dysfunction were excluded. On the other hand, review articles, systematic meta-analyses, case reports, unrelated articles, articles involving pre-existing thyroid diseases, studies focused on the general impact of COVID-19 on thyroid function in specific populations, and articles without full-text availability were excluded. In the second systematic review, the inclusion and exclusion criteria were the same as in the first systematic review. 

Data extraction: For each selected study, reviewers independently collected details including authors, publication year, study design, patient demographics (number of COVID-19 patients evaluated, mean age, sex percentage), pathology data [C-reactive protein (CRP), TFTs such as TSH, T3, T4 and antibodies alike anti-thyroid peroxidase (TPO), and anti-Tg], and potential clinical value. Key findings focused on TFT abnormalities in COVID-19 patients and the impact of vaccines on thyroid dysfunction. All studies included were based on patients with confirmed COVID-19 infection.

Quality assessment: The Newcastle-Ottawa Scale (NOS) was used to assess the quality of the case-control studies (10).

## 4. Results

### 4.1. Systematic Review on Thyroid Function in COVID-19 Patients and Its Relationship with the Severity of COVID-19 Infection

Among the 21 evaluated articles, seven studies investigated the COVID-19 positive and healthy controls patients (11-17) (Table 1), and 14 articles studied the severity of the infection on thyroid dysfunction (18-31) (Table 2); thyroid dysfunction was identified by abnormal thyroid test results, which varied across studies.

**Table 1.** Summary of Seven Selected Studies in COVID-19 Positive and Negative Groups

Authors	Study Type	Samples (COVID-19 Patients)	Thyroid Function Evaluation	Other Factors Evaluation	Results	Potential Clinical Value
**Khoo et al. (2020) (** 11 **)**	Cohort observational	Positive COVID-19: n = 334, negative COVID-19: n = 122	TSH, FT4	Albumin, CRP, cortisol, renal function	↓TSH, ↓FT4	Most patients with COVID-19 present with Euthyroidism
**Li et al. (2020) (** 12 **)**	Cross-sectional	Non-severe COVID-19 patients: n = 40, healthy controls: n = 57	FT3, TSH	GH, PTH, ALT, AST, Ca, CRP, CK, CK-MB, IL-1β, IL-8	↓TSH, ↓FT3	SARS-CoV-2 infection can affect, thyroid and parathyroid glands
**Okwor et al. (2021) (** 13 **)**	Cross-sectional	COVID-19: n = 45, healthy controls: n = 45	FT3, FT4, TSH	hs-CRP	↑FT3, ↑TSH	Higher TSH and FT3 levels in SES and subclinical hypothyroidism noted among some COVID-19 patients could be due to adaptive changes, intrinsic thyroid gland abnormalities, or damage to the hypothalamic-pituitary-thyroid function.
**Lui et al. (2021) (** 14 **)**	Prospective	COVID-19: n = 79, non-COVID-19: n = 44	TSH, FT4, FT3, anti-Tg, anti-TPO	HbA1c, CRP	NS	SARS-CoV-2 viral, load in acute COVID-19 inversely correlated with the thyroid volume, especially in men
**Malik et al. (2021) (** 15 **)**	Retrospective	COVID-19 positive: n = 48, COVID-19 negative: n = 28	TT3, TT4, TSH	IL-6, Procalcitonin	↑TSH and ↑TT3	Solid evidence of the high risk of altered thyroid function after COVID-19 pneumonia have provided.
** Hadisi et al. (2022) (** 16 **)**	Cross-sectional	COVID-19 positive: n = 60, healthy controls: n = 60	TSH	FSH, LH, prolactin, progesterone, testosterone, cortisol	↓TSH	COVID-19 affected directly or indirectly glands and related hormones.
**Wang et al. (2021) (** 17 **)**	Retrospective	COVID-19 positive: n = 84, non-COVID-19 pneumonia: n = 91, healthy controls: n = 807	TT3, TT4, TSH,	CRP, Procalcitonin, leukocytes, IL-6, IL-10, TNF-a, Interferon-γ	↓TT3 and ↓TSH, ↓TT4	Thyroid function abnormalities are common in COVID-19 patients, especially in severe cases. This may be partially explained by NTIS, it is also possible that the thyroid gland is a direct target of the SARS CoV-2 virus.

Abbreviations: A1C, glycated hemoglobin; ALT, alanine aminotransferase; Anti-TSHR, anti-TSH receptor antibody; AST, aspartate aminotransferase; CK, creatine kinase; Cr, creatinine; CREA, creatine kinase; cTnI, cardiac troponin I; D2D, D dimer; ESR, erythrocyte sedimentation rate; SES, sick euthyroid syndrome; FSH, follicle-stimulating hormone; FT3, free triiodothyronine; FT4, free thyroxine; HRCT, high resolution computed tomography; hs-CRP, high sensitive C-reactive protein; hsTNI, high sensitive troponin I; ICU, intensive care unit; IL-6, Interleukin-6; IQR, inter quartile range; LDH, lactate dehydrogenase; LH, luteinizing hormone; MYO, myohemoglobin; NS, not significant; NSAIDs, nonsteroidal anti-inflammatory drugs; NR, not reported; TFTs, thyroid function tests; NTIS, non-thyroidal illness syndrome; NT-proBNP, N-terminal prob-type natriuretic peptide; PCT, procalcitonin;; pro-BNP, pro-brain natriuretic peptide; qRT-PCR, quantitative reverse transcriptase PCR; SCr, serum creatinine; SD, standard deviation; sICU, sub-intensive care unit; TBil, total bilirubin; TD, thyroid dysfunction; Tg, thyroglobulin; TgAb, thyroglobulin antibodies; TH, thyroid hormones; TNF, tumor necrosis factor; TPOAb, thyroperoxidase antibody; TRAb, TSH receptor antibodies; TSH, thyroid stimulating hormone; UREA, urea nitrogen, NS, not significant.

**Table 2.** Summary Results of the Literature Review in 14 Selected Studies with the Severity of COVID-19

Authors	Study Type	Samples (COVID-19 Patients)	Age (y)	Thyroid Function Evaluation	Other Factors Evaluation	Results	Potential Clinical Value
**Lui et al. (2021) (** 18 **)**	Prospective	Mild: n = 161, moderate: n = 24, severe: n = 6	Mean ± SD, 53.5 ± 17.2	TSH, fT4, fT3, fT3/fT4 ratio, Anti-TPO, Anti-Tg, anti-TSHR	CRP, ESR	↓SARS-Cov-2 Ct value with ↓TSH & ↓fT3 & ↓fT3/fT4	Low fT3, associated with systemic inflammation, may have a prognostic significance.
**Chen et al. (2020) (** 19 **)**	Retrospective	COVID-19: n = 50, moderate: n = 15, severe: n = 23, critical: n = 12, healthy Control: n = 54, non-COVID-19: n = 50	Mean ± SD, 48.4 ± 13.7	TT4, TT3, TSH, FT3, FT4	Albumin	↓TSH, ↓TSH and ↓TT3 in COVID-19 & severe COVID-19, TT4 in COVID-19 no change	The changes in serum TSH and TT3 levels may be important manifestations of the courses of COVID-19.
**Sen et al. (2021) (** 20 **)**	Cross-sectional	Mild: n = 26, moderate: n = 16, severe: n = 18	≥ 18	TT3, FT3, TT4, FT4, TSH, anti‑TPOAb	Ferritin	↓FT4	Some form of thyroid function alteration was seen in about one-third of patients during COVID-19 irrespective of the severity of the disease.
**Dabas et al. (2021) (** 21 **)**	Cross‑sectional	Mild: n = 22, moderate: n = 78, severe: n = 64	Mean ± SD 53.85 ± 19.54	fT4, fT3, TSH	IL-6	↓fT3 in sever COVID-19	Low fT3 was associated with death and increased inflammation, suggesting poor prognosis.
**Beltrao et al. (2021) (** 22 **)**	Prospective cohort	Critical: n = 64, non-critical: n = 181 =	62 (IQR: 49 - 74.5)	TSH, fT3, fT4, rT3, Tg, anti TPOAb	ALT, AST, creatinine, CRP, D-dimer, LDH, IL-6, ferritin	↑rT3 & ↓fT3 + high rT3 are lower frequent in critical groups	Serum TH levels were correlated with illness severity, mortality, and other biomarkers of critical illness.
**Clarke et al. (2021) (** 23 **)**	Prospective, observational	Mild: n = 12, moderate: n = 30, severe: n = 21, critical: n = 7	Mean ± SD, 55.9 ± 13.0	TSH, fT4, fT3	Cortisol, ACTH	TSH, fT4, fT3 within the normal range after 3 months	There was no increase in hypo- or hyperthyroidism frequency.
**Guven and Gultekin (2021) (** 24 **)**	Prospective	Mild symptoms in non-ICU (group1): n = 125 =, critical condition in ICU (group2): n = 125, survivor: n = 88, deceased: n = 37	Median (IQR), 68 (54-78)	TSH, FT4, FT3, Anti-TPO, Anti-Tg	Glucose, urea, creatinine, AST, ALT, albumin, ferritin, CRP, D-dimer	TSH not differ, ↓FT4 & ↓FT3,, Anti-TPO & anti-Tg overt hypothyroidism were ≥3 times higher	COVID-19 affects thyroid hormone metabolism, with low FT3 levels at admission indicating a higher risk of severe cases and poor prognosis. Thyroid nodules may also be a comorbidity that increases COVID-19 risk.
**Baldelli et al. (2021) (** 25 **)**	Retrospective	Hospitalized for COVID-19 pneumonia (A): n = 23, patients requiring ICU (B): n = 23, euthyroid (C): n = 20	Mean ± SD, A)60.8 ± 17.0, B)58.4 ± 12.1, C)59.1 ± 10.7,	FT3, FT4, TSH, AbTg, AbTPO	-	(1 ↓fT3 & ↓TSH in A & B compared to C, (2) ↓fT3 & ↓TSH in B compared to A	COVID-19 can cause NTIS and potential direct damage to the thyroid (and possibly pituitary) gland.
**Vassiliadi et al., (2021) (** 26 **)**	Prospective	COVID-19 positive: n = 102, admitted in the ICU (ICU C+): n = 41, admitted in the ward (Ward C+): n = 46, outpatient C+: n = 15, control group: n = 94, admitted in the ICU (ICU NC): n = 39, admitted in the ward (Ward NC): n = 55	Mean ± SD, 63.0 ± 10.2, 53.8 ± 17.4, 38.9 ± 11.4, 56.7 ± 20.0, 69.3 ± 17.8	T3, FT4, TSH anti-TPO, anti-Tg, Tg	IL-6	↑FT4 in ICU C+	NTIS pattern is common and relates to the severity of disease.
**Ahn et al. (2021) (** 27 **)**	Retrospective cohort	Non-severe: n = 32, Severe to critical: n = 87	Mean ± SD, 64.3 ± 16.8	T3, FT4, TSH	CRP	↓T3, ↓TSH	NTIS was frequently seen in patients with severe baseline manifestations.
**Lui et al. (2021) (** 28 **)**	Prospective	Symptomatic acute COVID-19: n = 172, asymptomatic acute COVID-19: n = 32	Median (IQR), 55, (44.3-63.0)	TSH, fT4, fT3, anti-Tg, anti-TPO	HbA1C, CRP	↓fT3, ↑anti-TPO	Most abnormal thyroid function tests in acute COVID-19 resolved, with rare cases of incident thyroid dysfunction.
**Gao et al. (2021) (** 29 **)**	Retrospective	Non-severe: n = 34, severe or critical: n = 66	Mean ± SD, 62.3 ± 14.3	FT3, FT4, TSH, FT3/FT4	IL-6, TNF-α, NT-proBNP, D-dimer, ALT, AST, FBS, total bilirubin, direct bilirubin, albumin, creatinine	↓FT3, ↓TSH, ↓FT3/FT4	The reduced FT3 independently predicts all-cause mortality of patients with severe COVID-19 and it may become a simple tool for the stratified management of patients with severe COVID-19.
**Kumar, et al (2021) (** 30 **)**	Cross-sectional	Asymptomatic: n = 109, mild: n = 70, moderate: n = 23, severe: n = 22, critical: n = 11	Mean ± SD, 48.9 ± 16.4	FT4, FT3, TSH	Prolactin, Cortisol, IL‑6, hs-CRP	↓TSH & ↓FT3	Adrenal insufficiency, low T3 and low TSH syndrome, and hyperprolactinemia were common in hospitalized COVID-19 patients.
**Lang et al. (2021) (** 31 **)**	Retrospective	Survivors: n = 116, non-survivors: n = 11	Median (IQR), 66.0, (53.0–71.0)	TSH, FT3, FT4	D-dimer, IL 6, hs-cTnI, Creatinine, HbA1c	↓TSH, ↓FT3	Low FT3 state on admission was associated with an increased risk of all-cause in-hospital mortality in patients with COVID-19.

Abbreviations: A1C, glycated hemoglobin; ALT, alanine aminotransferase; Anti-TSHR, anti-TSH receptor antibody; AST, aspartate aminotransferase; CK, creatine kinase; Cr, creatinine; CREA, creatine kinase; cTnI, cardiac troponin I; D2D, D dimer; ESR, erythrocyte sedimentation rate; SES, sick euthyroid syndrome; FSH, follicle-stimulating hormone; FT3, free triiodothyronine; FT4, free thyroxine; HRCT, high resolution computed tomography; hs-CRP, high sensitive C-reactive protein; hsTNI, high sensitive troponin I; ICU, intensive care unit; IL-6, Interleukin-6; IQR, inter quartile range; LDH, lactate dehydrogenase; LH, luteinizing hormone; MYO, myohemoglobin; NS, not significant; NSAIDs, nonsteroidal anti-inflammatory drugs; NR, not reported; TFTs, thyroid function tests; NTIS, non-thyroidal illness syndrome; NT-proBNP, N-terminal prob-type natriuretic peptide; PCT, procalcitonin; pro-BNP, pro-brain natriuretic peptide; qRT-PCR, quantitative reverse transcriptase PCR; SCr, serum creatinine; SD, standard deviation; sICU, sub-intensive care unit; TBil, total bilirubin; TD, thyroid dysfunction; Tg, thyroglobulin; TgAb, thyroglobulin antibodies; TH, thyroid hormones; TNF, tumor necrosis factor; TPOAb, thyroperoxidase antibody; TRAb, TSH receptor antibodies; TSH, thyroid stimulating hormone; UREA, urea nitrogen.

In Table 1, the lowest sample size was 76 (15) and the highest was 982 for COVID-19 and controls (17). In three studies, the levels of TSH, T3, and T4 were reduced (11, 12, 16, 17), and in two studies the TSH and T4 or T3 significantly elevated (13, 15) and one study did not find a significant difference (14) in COVID-19 positive patients compared to the negative group. Results showed that COVID-19 patients exhibited abnormal thyroid function test compared to healthy controls. 

In Table 2, a minimum sample size of 60 (20) and a maximum of 250 (24) for severe COVID-19, considering those aged ≥ 20 years old in both genders were shown. One study did not report the number of males and females (12). Seven studies showed that the TSH levels were decreased in severe patients of COVID-19 (18, 19, 25, 27, 29-31); however, in two studies they did not report significant differences (23, 24). Approximately, all studies indicated the diminished levels of free triiodothyronine (fT3) or total T3 (TT3) in severe subjects compare to others; only two studies showed no significant changes in T3 levels among COVID-19 patients (23, 26), while in one study by Beltrao et al. (2021) in accompaniment to reduction of fT3, the level of reverse T3 (rT3) was raised (22). The fT3 to free thyroxine (fT4) ratio was decreased in two studies (18, 29), and furthermore the level of fT4 was significantly lower in severe patients in two studies (20, 24); and in another one study, its level was higher in severe patients compare to non-severe COVID-19 ones (26). In other studies, the fT4 levels were not significantly changed (18, 19, 21-23, 25, 27, 28, 31) Some studies also found increased levels of anti-thyroid peroxidase (anti-TPO) and anti-thyroglobulin (anti-Tg) antibodies in COVID-19 patients (24, 28). Findings indicated that the severity of COVID-19 infection appeared to correlate with the degree of thyroid dysfunction, with more severe cases showing greater abnormalities in TFTs. 

### 4.2. Systematic Review on the Effect of COVID-19 Vaccines on Thyroid Function

Data on regions, samples, and vaccine types, duration of follow-ups, age, sex, TFTs evaluation, results, and potential outcomes from the five studies investigating the impact of COVID-19 vaccination on thyroid function are presented in Table 3. Among five selected studies (32-36), the highest sample size was 2,288,239 people (33), and the lowest was 70 subjects (32). All studies were among adults aged ≥ 18 years. In two studies, CoronaVac and BNT162b2 were evaluated (33, 34) and in two other studies only BNT162b2 mRNA vaccine was studied (35, 36). One study did not mention the type of vaccine (32). This limited evidence recommends that COVID-19 vaccines may be associated with some individuals developing or intensifying thyroid dysfunction and autoimmunity.

**Table 3.** Summary Results of the Literature Review in 5 Selected Studies of the Impact of COVID-19 Vaccination on Thyroid Function

Authors	Samples (COVID-19 Patients)	Vaccines	Follow-up	Age (y)	Thyroid Function Evaluation	Results	Potential Clinical Value
**Razu et al. (2022), (** 32 **)**	Healthy (A): n = 10, COVID-19 (+) unvaccinated (B): n = 30, COVID-19 (-) vaccinate (C): n = 30	NR	5 months	Mean ± SD 35.30 ± 15.60	TT3, TT4, TSH	↑TSH levels in both group B and C than in the control group A, while the ↓TT3 and ↓TT4 levels lower in both groups compared to the healthy controls.	Abnormalities in thyroid function can happened during COVID-19 infection and after vaccination.
**Wong et al. (2022), (** 33 **)**	Total: 2,288,239	Inavtivated (CoronaVac) and mRNA (BNT162b2)	56 days	Aged ≥ 18	TSH	NS	No evidence observed of vaccine-related increase in incident hyperthyroidism or hypothyroidism with both BNT162b2 and CoronaVac.
**Lui et al. (2022), (** 34 **)**	BNT162b2 recipients: n = 129, CoronaVac recipients: n = 86	Inavtivated (CoronaVac) and mRNA (BNT162b2)	8 weeks	Mean ± SD 49.6 ± 12.5	TSH, FT3, FT4, anti-TPO, anti-Tg	↑fT4, ↓fT3, ↓T3/T4, ↑anti-Tg, and ↑anti-TPO among both vaccine recipients, and ↑fT4 and ↑TSH only among CoronaVac recipients	COVID-19 vaccination was associated with a modest increase in antithyroid antibodies but did not cause clinically significant thyroid dysfunction 8 weeks after vaccination.
**Paschou et al. (2022), (** 35 **)**	Autoimmune thyroiditis: n = 56 Healthy controls: n = 56	mRNA (BNT162b2)	3 months (2 doses)	Median 51	Positive anti-TPO or/and anti-TG	Neutralizing antibodies (NS)	Patients with autoimmune thyroiditis present similar immunological response to COVID-19 BNT162b2 mRNA vaccine with healthy subjects.
	Total: 72 healthy		1 month, (2 doses)	Median 45	T3, T4, TSH, anti-Tg, anti-TPO	↓T3 and ↓TSH	Vaccination may affect thyroid function, namely decrease TSH and T3 levels.
**Morita et al. (2023), (** 36 **)**	Total: 90	mRNA (BNT162b2)	12 month (3 doses)	Median 50 (IQR, 38-54)	TRAb, TgAb, TPOAb, TSH, FT4 and FT3	↑TRAb and ↑TgAb, THs (NS)	SARS-CoV-2 BNT162b2 mRNA vaccine can disrupt thyroid autoimmunity.

Abbreviations: A1C, glycated hemoglobin; ALT, alanine aminotransferase; Anti-TSHR, anti-TSH receptor antibody; AST, aspartate aminotransferase; CK, creatine kinase; Cr, creatinine; CREA, creatine kinase; cTnI, cardiac troponin I; D2D, D dimer; ESR, erythrocyte sedimentation rate; SES, sick euthyroid syndrome; FSH, follicle-stimulating hormone; FT3, free triiodothyronine; FT4, free thyroxine; HRCT, high resolution computed tomography; hs-CRP, high sensitive C-reactive protein; hsTNI, high sensitive troponin I; ICU, intensive care unit; IL-6, Interleukin-6; IQR, inter quartile range; LDH, lactate dehydrogenase; LH, luteinizing hormone; MYO, myohemoglobin; NS, not significant; NSAIDs, nonsteroidal anti-inflammatory drugs; NR, not reported; TFTs, thyroid function tests; NTIS, non-thyroidal illness syndrome; NT-proBNP, N-terminal prob-type natriuretic peptide; PCT, procalcitonin;; pro-BNP, pro-brain natriuretic peptide; qRT-PCR, quantitative reverse transcriptase PCR; SCr, serum creatinine; SD, standard deviation; sICU, sub-intensive care unit; TBil, total bilirubin; TD, thyroid dysfunction; Tg, thyroglobulin; TgAb, thyroglobulin antibodies; TH, thyroid hormones; TNF, tumor necrosis factor; TPOAb, thyroperoxidase antibody; TRAb, TSH receptor antibodies; TSH, thyroid stimulating hormone; UREA, urea nitrogen.

## 5. Discussion

This review examines the impact of COVID-19 on thyroid health from multiple angles. It explores thyroid function in both individuals who have contracted COVID-19 and those who have not, providing a comparative analysis. Additionally, the virus's effects on THs were investigated, as well as the development of thyroid disorders during the illness. This review also delves into the potential connections between COVID-19 and NTIS, a condition characterized by abnormal TFTs in the absence of primary thyroid disease. Furthermore, it addresses the possibility of vaccine-induced thyroid inflammation, a crucial consideration in the ongoing efforts to combat the pandemic.

### 5.1. Systematic Review on Thyroid Function in COVID-19 Positive and Negative Patients 

Seven studies have shown various levels of thyroid dysfunction in patients infected with COVID-19 compared to non-infected individuals (Table 1). In a study by Khoo et al., patients with COVID-19 exhibited lower levels of TSH and FT4 compared to those without COVID-19. However, the majority of COVID-19 patients presented with euthyroid status, with no cases of overt thyrotoxicosis (11). Li et al. analyzed thyroid and parathyroid gland markers in non-severe COVID-19 patients versus healthy controls. They also found reduced levels of TSH and FT3, along with increased levels of PTH in the COVID-19 patients compared to the control group (12). Hadisi et al. found that men infected with COVID-19 had lower TSH levels than controls (16). The abnormal TSH levels in COVID-19 patients may indicate negative feedback related to T3 and T4 levels, suggesting possible thyroid gland injury. Furthermore, Wang et al. found lower TT3 and TSH levels in COVID-19 patients compared to healthy controls, as well as lower levels of TT4 in non-COVID-19 pneumonia patients compared to those with COVID-19. The TT3-TSH correlation was initially positive but became negative over time (17). Thyroid dysfunction is common in COVID-19, especially in severe cases, potentially due to NTIS. These disparities highlight the complex and diverse thyroid-related manifestations observed in patients with COVID-19. However, Lui et al. found no significant differences in TH levels between patients and controls, but ultrasonography showed that higher SARS-CoV-2 viral loads at presentation were linked to smaller thyroid volumes, especially in men (14). Most COVID-19 patients showed normal thyroid function; however, the suppression of TSH is most likely related to elevations in pro-inflammatory cytokines such as IL-6, which are negatively correlated with TSH (37). 

In contrast, Okwor et al. reported significantly higher plasma FT3 and TSH concentrations in COVID-19 patients compared to controls (13). Moreover, Malik et al. indicated that TSH was elevated in COVID-19 patients with moderate and critical illness compared to non-COVID-19 patients. Additionally, during the follow-up period, COVID-19 patients showed a significant increase in TT3 levels, while TT4 levels remained relatively unaffected (15). Elevated levels of TSH and FT3, along with SES and subclinical hypothyroidism, indicate a potential link between the disease and thyroid abnormalities. Therefore, it is important to monitor thyroid function both during and after COVID-19 treatment. Additionally, elevated TSH levels may be influenced by the timing of sample collection, as a mild and temporary rise in TSH can occur during recovery from SES. In classic SES, T3 and TSH levels are usually reduced, while severe cases may also see a decrease in T4 levels (38).

### 5.2. Systematic Review on Thyroid Function Relationship with the Severity of COVID-19 Infection 

Fourteen studies evaluated the impact of COVID-19 severity on thyroid dysfunction (Table 2). Lui et al. reported that patients with mild to moderate infections exhibited thyroid dysfunction, noting that a lower cycle threshold (Ct) value, indicating higher viral RNA load, was the only independent factor linked to reduced TSH and fT3 levels (18). Several studies have associated thyroid disorders with severe COVID-19 outcomes and poor prognosis (21, 22, 27, 39). Chen et al. found a positive correlation between decreased TSH and total T3 levels and COVID-19 severity, suggesting direct effects on TSH-secreting cells in the pituitary (19). Sen et al. noted altered thyroid function, particularly low fT4 levels, but a clear pattern of dysfunction was not established in most patients, likely due to the coexistence of SAT and SES, resulting in mixed presentations (20). Dabas et al. reported SES with low fT3 and severe inflammation (increased IL-6) (21). Beltrao et al. found decreased serum fT3 and increased rT3 levels in COVID-19 patients without clinical symptoms of SAT and NTIS (22). Guven and Gultekinreported lower FT4 and FT3 levels in severe patients, while TSH levels did not differ between critical ICU patients and mild non-ICU patients (24). Baldelli et al. showed significant reductions in fT3 and TSH in hospitalized COVID-19 patients compared to euthyroid controls (25). Ahn et al. observed that declines in TSH and T3 levels were significantly correlated with COVID-19 severity (27), and Lui et al. reported lower fT3 levels in symptomatic acute COVID-19 patients (28). Gao et al. found that FT3, TSH, and FT3/FT4 levels decreased with clinical deterioration, with lower FT3 levels linked to higher mortality in severely ill patients (29). Changes in TSH levels may result from the indirect effects of viral infection activating pro-inflammatory cytokines. Kumar et al. and Lang et al. reported decreased TSH and FT3 levels, with lower levels in asymptomatic patients compared to severe cases and in non-survivors versus survivors (30, 31). However, Clarke et al. found that patients without preexisting thyroid disease had TSH, fT4, and fT3 levels within the reference range at least three months after COVID-19 presentation (23). Higher anti-TPO levels accompanied by lower fT3 and fT4 levels were reported in severe patients (24, 28), indicating that COVID-19 and the associated cytokine storm may disrupt the HPT axis.

In contrast, Vassiliadi et al. reported marginally higher FT4 levels in ICU SARS-CoV-2 positive patients than in ICU negative patients (26). This increase in T4 may relate to measurement accuracy, which in this study correlated with the severity of thyroid disorders. T4 measurements can be prone to errors and may not always be accurate.

### 5.3. Mechanisms Involved in COVID-19 Infection and Thyroid Dysfunction 

SARS-CoV-2 can affect the thyroid both directly and indirectly through immune dysregulation. Increased levels of anti-thyroid antibodies (anti-TPO and anti-Tg) were reported, with worsened clinical severity, elevated CRP, and higher initial antibody titers linked to significant increases in anti-TPO levels (40). This suggests that COVID-19's hyper-inflammatory state, primarily driven by Th1 cells and IL-6, may promote autoimmunity. Other studies have noted elevated IL-6 during destructive thyroiditis and increased activation of Th1-like peripheral lymphocytes in autoimmune thyroiditis (41, 42). These findings indicate that SARS-CoV-2 can induce transient and reversible thyroid dysfunction, potentially through direct viral effects or immune-mediated mechanisms. Monitoring thyroid function in COVID-19 patients is important, as dysfunction can impact disease progression and recovery. However, the included studies in this review were mostly observational, with small sample sizes and heterogeneity in patient populations, COVID-19 severity, and timing of TFTs. The long-term consequences of COVID-19-associated thyroid dysfunction remain unclear.

Cytokine storms, characterized by excessive release of inflammatory cytokines like IL-1β, TNF-α, IL-6, and IFN-γ, are associated with severe COVID-19 and can suppress the HPT axis, leading to decreased TSH secretion (43, 44). This suppression involves central regulation, hormone production, and receptor activity (45). Studies indicate that the decrease in TSH during COVID-19 is transient, with TFTs returning to baseline during follow-up. The cytokine storm can induce NTIS, affecting TSH levels and reducing the conversion of FT4 to FT3 (11, 46). A slight decrease in serum FT4 and TSH levels was observed in 13% of 383 COVID-19 patients, consistent with NTIS, and increased pro-inflammatory cytokines, particularly IL-6, are negatively correlated with TSH levels (11).

In severe COVID-19, the cytokine storm results from a hyperactive Th1/Th17 immune response, leading to elevated IL-6 and TNF-α (47). Elevated rT3 levels typically result from slower clearance and accelerated deiodination of T4 (48). Deiodinases play a crucial role in NTIS, causing decreased T3 in critically ill patients, and SARS-CoV-2-triggered cytokine expression significantly contributes to NTIS (49). Non-thyroidal illness syndrome may occasionally present with thyrotoxicosis, especially in patients with low FT3 and high FT4, suggesting that viral infection could trigger autoimmunity (50).

### 5.4. Systematic Review on the Effect of COVID-19 Vaccines on Thyroid Function 

Five studies with follow-up periods ranging from 1 to 12 months have investigated the impact of COVID-19 vaccination on thyroid function (Table 3). The available reports on the effects of vaccination on THs present conflicting results. Razu et al. conducted a study with a five-month follow-up and found that unvaccinated COVID-19 positive patients and vaccinated COVID-19 negative patients exhibited higher TSH levels and lower TT4 and TT3 levels compared to healthy controls. However, the levels were similar between the two patient groups (32). Wong et al. found no significant changes in thyroid hormone levels before and after vaccination following a follow-up period of 56 days after the first and second doses (33). 

Morita et al. observed that the BNT162b2 vaccine did not significantly alter thyroid hormone levels. However, they found evidence of increased TRAb and TgAb levels, which are markers of thyroid autoimmunity. The researchers suggested that the post-vaccination increases in TSH receptor and thyroglobulin antibodies might predict the development of autoimmunity, particularly in females with a history of autoimmune thyroid disease. Furthermore, low pre-vaccination FT4 and FT3 levels, along with prior thyroid disease markers, were associated with elevated antibody levels after the third vaccine dose. These findings indicate the potential for vaccine-induced autoimmunity (36). 

While these three studies reported no changes in thyroid hormone levels before and after vaccination, Lui et al. observed different results. After the second dose of both vaccines, they reported higher levels of fT4, anti-Tg, and anti-TPO, along with lower fT3 levels and a decreased T3/T4 ratio after two months. Specifically, patients who received the BNT162b2 vaccine had significantly lower fT3 and T3/T4 ratios, while exhibiting higher anti-TPO and anti-Tg levels post-vaccination. In contrast, those who received the CoronaVac vaccine showed higher TSH, fT4, anti-Tg, and anti-TPO levels, along with lower fT3 and T3/T4 ratios post-vaccination (34). Although this study indicated lower TSH levels and higher fT4 levels after receiving the mRNA-based vaccine, these changes were not statistically significant. On the other hand, Paschou et al. found decreased T3 and TSH levels one month after BNT162b2 vaccination, but reported no significant changes in antibody titers or overt hypothyroidism (35). Although in the study by Lui et al., there was no significant decrease in TSH levels observed 8 weeks after receiving the second dose of the BNT162b2 mRNA vaccine, Paschou et al. (34, 35). reported a significant decrease in TSH levels 4 weeks after the administration of the same vaccine. This discrepancy may be attributed to the possibility that TSH levels decrease significantly shortly after vaccination (around 4 weeks) but tend to return closer to normal levels over a longer period (8 weeks) (51).

One theory is that a systemic immune-mediated inflammatory response after vaccination affects the thyroid gland, reducing T3 levels, and/or influences the pituitary gland, leading to lower TSH levels and indirectly affecting thyroid function. Another explanation could be an underlying NTIS, often seen in illnesses, characterized by normal or low TSH and low T3 levels, with normal or low T4 concentrations. This reflects the body's adaptive mechanism to recover from illness (52).

### 5.5. Potential Mechanisms of Thyroid Dysfunction Following COVID-19 Vaccination 

Research on the effects of COVID-19 vaccines on THs is still emerging, yet evidence suggests that vaccination can affect thyroid function, while the mechanisms behind the development of thyroid autoimmunity following the SARS-CoV-2 mRNA vaccine remain debated. 

Two primary theories have emerged: The induction of autoimmune/inflammatory syndrome induced by adjuvants (ASIA) and molecular mimicry, where vaccine-encoded proteins may interact with the angiotensin-converting enzyme II (ACE-2) receptor, cross-react with thyroid self-proteins, and trigger immune reactions from adjuvants (53-55). ASIA occurs in genetically susceptible individuals when adjuvants disrupt immune balance, leading to post-vaccination autoimmune phenomena. While adjuvants are designed to enhance immune responses to pathogens, some can trigger adverse reactions, as shown in animal studies (54). The RNA-based vaccines utilize adjuvants, such as lipid nanoparticles, which may provoke an exaggerated immune response and contribute to thyroid autoimmunity (56).

Reports from healthcare professionals indicate rare cases of thyroiditis following vaccination (57, 58), with instances of autoimmune and inflammatory thyroid diseases, such as SAT, Graves' disease, and chronic autoimmune thyroiditis, noted after COVID-19 vaccination, though causality remains debated (59, 60). Subacute thyroiditis typically arises from thyroid inflammation due to viral infections, with adenovirus and Epstein-Barr virus identified as potential triggers (61). Two cases of vaccine-induced Graves' disease were reported in female healthcare workers who exhibited thyrotoxicosis symptoms 2 - 3 days after receiving the Pfizer-BioNTech vaccine (62). A systematic review concluded that thyroid disease onset occurred, on average, 11 days post-vaccination (63).

While the exact mechanisms behind vaccination-induced thyroid changes are unclear, they may affect susceptible individuals through direct viral effects and inflammatory responses (49). Subacute thyroiditis has been reported following various vaccines, including Coronavac and AstraZeneca, as well as mRNA vaccines from BioNTech-Pfizer and Moderna (64-66). It is suggested that viral proteins may stimulate cytotoxic T lymphocytes, damaging thyroid follicular cells rich in ACE-2 receptors (67). Additionally, similarities between viral and human proteins, such as TPO, may trigger autoimmune responses (68). Although vaccine adjuvants enhance immunogenicity, evidence indicates they are unlikely to be the cause of COVID-19 vaccine-related SAT (69), particularly since mRNA vaccines, which do not contain traditional adjuvants, have been associated with SAT cases (21, 65, 70). RNA vaccines use lipid nanoparticles that may overstimulate the immune system (56, 71). Recent reports also suggest an increased risk of thyroid eye disease in individuals with a history of Graves' disease following COVID-19 vaccination, with isolated SAT cases noted, especially after mRNA vaccines (72-75). 

Despite these concerns, the benefits of vaccination outweigh the risks, and individuals with thyroid dysfunction are encouraged to be vaccinated, as they face a higher risk of severe COVID-19 complications. The American Thyroid Association advises these individuals to continue their regular thyroid medications and monitoring, as the vaccine is unlikely to interfere with TFTs (67). Monitoring is recommended for younger individuals at risk for autoimmunity during COVID-19 vaccination, and clinicians should keep an eye on thyroid function in patients exhibiting post-vaccination SAT or Graves' symptoms (54). Current limitations include a small number of studies with limited sample sizes, underscoring the need for larger, more robust studies to assess the risk of vaccine-induced thyroid dysfunction.

### 5.6. Conclusions

This systematic review found that COVID-19 patients often have abnormal thyroid function test results compared to healthy controls, including decreased levels of TSH, T3, and T4, along with increased anti-thyroid antibodies. The severity of COVID-19 infection correlates with the extent of thyroid dysfunction, suggesting that SARS-CoV-2 can induce transient thyroid issues, possibly through direct viral effects or immune response. Monitoring thyroid function in COVID-19 patients is important, as dysfunction can affect disease progression and recovery.

Limited evidence indicates that COVID-19 vaccines may be linked to the development or worsening of thyroid dysfunction and autoimmunity in some individuals, although the overall risk appears low and effects are generally transient. Patients with pre-existing autoimmune thyroid disorders may be at greater risk. Clinicians should be aware of these rare thyroid-related adverse effects and monitor thyroid function in high-risk patients. Continued research is necessary to better understand the relationship between COVID-19 vaccines and thyroid health.

## Data Availability

The dataset presented in the study is available on request from the corresponding author during submission or after publication.
